# Myocardial Bridging Unmasks as an Acute Coronary Syndrome from Dehydration

**DOI:** 10.1155/2021/5589776

**Published:** 2021-07-12

**Authors:** Naji Maaliki, Michael Omar, Aleem Azal Ali, Amy Roemer, Jose Ruiz, Edin Sadic

**Affiliations:** ^1^Internal Medicine, University of Florida College of Medicine, Jacksonville, USA; ^2^Cardiology, University of Florida College of Medicine, Jacksonville, USA

## Abstract

A 50-year-old male presented for loss of consciousness. He was initially treated with intravenous epinephrine and fluids, and an electrocardiogram (ECG) displayed an ST-segment elevation in lead aVR with global ST-segment depressions. A subsequent left heart catheterization revealed that the middle segment of the left anterior descending artery (LAD) demonstrated severe stenosis during systole but would become patent during diastole, which was suggestive of myocardial bridging. After stopping the epinephrine and increasing the fluid infusion, the ECG changes rapidly resolved. The patient had later admitted to significant dehydration all day. Myocardial bridging is a congenital anomaly in which a coronary artery segment courses through the myocardium instead of the usual epicardial surface. Occasionally, myocardial bridging may present similarly to acute coronary syndrome in severe dehydration or hyperadrenergic states. The diagnosis can be made through coronary angiography, which reveals a dynamic vessel obstruction pattern corresponding with the cardiac cycle. Long-term effects may also include accelerated atherosclerosis. Treatment consists of reversing precipitating causes during acute presentations and decreasing the risk of coronary artery disease on a chronic basis.

## 1. Introduction

New-onset ST-segment changes on electrocardiogram (ECG) are usually a cause of concern, with coronary artery occlusion among the lead differential diagnoses. Myocardial bridging is a peculiar condition that may lead to such changes, yet the vessel obstruction is mostly dynamic and dependent on the cardiac cycle. Though usually asymptomatic, it can manifest in emergent cases such as acute coronary syndrome when precipitated by some factors and long-term atherosclerosis.

## 2. Case Presentation

**Figure 1:**
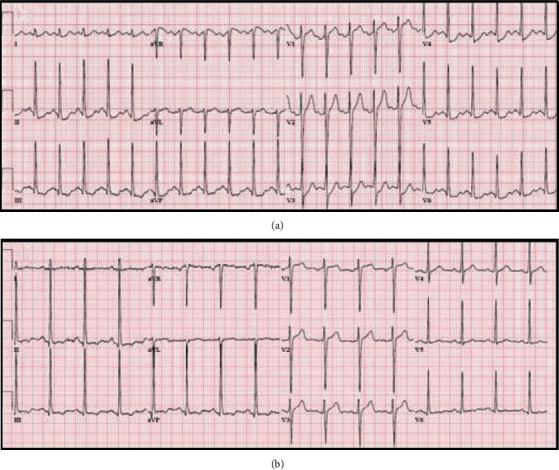
(a) Initial ECG displaying ST-segment elevation in lead aVR and diffuse ST-segment depressions. (b) ECG after aggressive hydration displaying normalization of ischemic changes.

**Figure 2:**
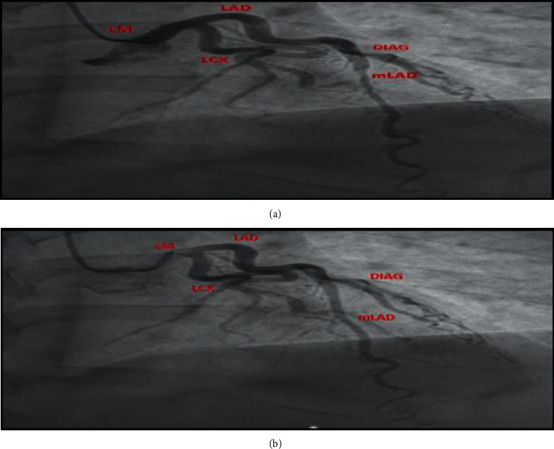
(a) RAO cranial LHC demonstrating significant middle-LAD stenosis during systole. (b) RAO cranial LHC demonstrating patent middle-LAD during diastole.

A 50-year-old male with a past medical history of hypertension presented for loss of consciousness. He was hypotensive with a mean arterial pressure of 55 mmHg, tachycardic with a heart rate around 140 bpm, and minimally arousable on initial evaluation while complaining of ill-defined chest pain. Physical exam showed a regular tachycardic rhythm, normal heart sounds, decreased bilateral air entry, and absent jugular venous distention or peripheral edema. Treatment started in the emergency department with intravenous fluids and epinephrine. The electrocardiogram (ECG) displayed an ST-segment elevation in lead aVR, along with global ST-segment depressions, most notably in the inferolateral leads (Figure 1(a)). Point of care troponin I was elevated at 0.42 ng/mL. An emergent left heart catheterization (LHC) revealed a smooth 99% filling defect at the middle segment of the left anterior descending artery (LAD) during systole (Figure 2(a)) that would rhythmically appear patent during diastole (Figure 2(b)), which was suggestive of myocardial bridging. The coronaries were otherwise angiographically normal. Epinephrine was discontinued, and after high-volume rehydration, blood pressure soon normalized. Follow-up ECG demonstrated a complete resolution of the ischemic changes (Figure 1(b)), and the patient regained consciousness. Postprocedure cardiac echocardiography showed moderate concentric hypertrophy with a normal global systolic function. On reevaluation, he claimed that he was dehydrated after heavy alcohol drinking in the sun. He also attested to having a few episodes of mild, exertional, left-sided chest pressure in the past that spontaneously resolved with rest and that he never sought any medical care for it. He was then started on carvedilol and a low-intensity statin with instructions to avoid dehydration and adrenergic agents.

## 3. Discussion

Myocardial bridging is a congenital anomaly in which a segment of the coronary arteries courses through the myocardium, thus rendering it “bridged” by the overlying cardiac tissue. While often benign, it may lead to emergent clinical presentations and long-term vascular disease [1]. Myocardial bridging most commonly affects the middle segment of the LAD. The diagnosis can be made using coronary angiography, which demonstrates a characteristic “milking” obstruction of the bridged vessel [2]. The myocardial compression of the artery decreases anterograde blood flow during systole and can even extend into early diastole. During tachycardia, diastole becomes compromised, leading to a shortened time for myocardial relaxation; thus, a higher proportion of flow obstruction can be seen within the cardiac cycle. Additionally, increased inotropy can exacerbate the degree of vessel compression, further prolonging the obstructive effect [3]. These manifestations may become clinically significant in hyperadrenergic states such as dehydration, anemia, and the administration of adrenergic compounds. Long-term atherosclerosis has also been noted in the segment proximal to the bridge. Due to the increased shear wall stress and angulation at the bridged portion, there is increased flow turbulence in the upstream segment [4].

The treatment of myocardial bridging involves both avoiding the acute limitation of blood flow and the long-term decrease in symptoms and atherosclerosis incidence. Patients should refrain from conditions that may induce an adrenergic drive and dehydration. Beta-blockers have been the cornerstone of therapy as they increase the diastolic phase and decrease inotropy, in addition to nondihydropyridine calcium-channel blockers as an adjunct therapy or when beta-blockers are not tolerated [5]. As discussed above, hydration may be beneficial when hypovolemia is suspected. Conversely, nitroglycerin is contraindicated as it can cause reflex tachycardia and sympathetic response [5, 6]. When there is concurrent atherosclerosis, aspirin and statins are used to prevent accelerated atherosclerosis [1]. In symptomatic cases refractory to maximal medical therapy, surgical management with coronary artery bypass graft (CABG) or myotomy surgeries can be considered [2, 5, 6]. CABG commonly involves grafting the left internal mammary artery to the LAD, and results have shown substantial symptom relief, with the main complication being graft failure [5]. Myotomy is done by dissecting the muscle fibers surrounding the bridged vessel portions, but complications include ventricular wall perforation, uncontrolled bleeding, and ventricular aneurysm formation [5]. While limited reports have shown symptomatic relief with both techniques, further studies are needed to delineate each option's associated risks and benefits and compare outcomes [2, 5].

## 4. Conclusion

This case highlights the importance of identifying myocardial bridging as a potential cause of myocardial ischemia, especially in patients presenting with dehydration and hyperadrenergic states. Early recognition may guide proper treatment and avoid disease exacerbation.

## Abbreviations

ECG: Electrocardiogram
LAD: Left anterior descending artery
LHC: Left heart catheterization
CABG: Coronary artery bypass grafting.

## Additional Points

*Limitations*. Limitations of this case report include the lack of a control group for comparison, one study subject indicating a low sample size, and lack of long-term follow-up.
